# Breast cancer in Angola, molecular subtypes: a first glance

**DOI:** 10.3332/ecancer.2017.763

**Published:** 2017-08-30

**Authors:** Fernando Miguel, Lygia Vieira Lopes, Eduardo Ferreira, Emília Ribas, Alexis Fuentes Pelaez, Conceição Leal, Teresina Amaro, Paula Lopes, Cristina Mendes Santos, Carlos Lopes, Lúcio Lara Santos

**Affiliations:** 1 Angolan Institute of Cancer Control, Rua Amílcar Cabral, Luanda, Angola; 2 Sagrada Esperança Clinic, Av Murtala Mohammed, Luanda, Angola; 3 Pathology Service, Portuguese Institute of Oncology, Rua António Bernardino de Almeida, Porto 4200-072, Portugal; 4 Eduardo Ferreira Pathology Laboratory, Rua de Camões, 218-5º andar, Porto 4000-000, Portugal; 5 Experimental Pathology and Therapeutics Group, Portuguese Institute of Oncology, Rua António Bernardino de Almeida, Porto 4200-072, Portugal; 6 ONCOCIR – Education and Care in Oncology – Lusophone Africa, Rua de Quires 168-10J, Moreira da Maia 4470- 643, Portugal; 7 Surgical Oncology Department, Portuguese Institute of Oncology, Rua António Bernardino de Almeida, Porto 4200-072, Portugal; * These authors contributed equally to this work.

**Keywords:** breast carcinoma, Angola, hormone receptors, molecular subtypes

## Abstract

**Purpose:**

The aim of this study was to investigate the breast cancer (BC) molecular subtypes according to its surrogate immunohistochemistry (IHC) markers. We conducted a preliminary study, to correlate the clinical pathological profiles and molecular subtypes of breast cancer in Luanda, Angola.

**Methods:**

From January 2011 to 30 December 2014, 140 consecutive cases of microscopically confirmed invasive breast carcinoma were classified regarding histology and IHC (ER, PR, HER2, and Ki-67). Surrogate molecular subtypes were classified according to ESMO recommendations.

**Results:**

All patients were female; the median age was 47 years (24–84 years). Invasive carcinoma NST was the most common type (91.4%) and grade 2 was prevalent (70.7%). Most tumours were locally advanced (stage III – 65% and stage IV – 3.6%). In 140 studied cases, 74 (52.8%) malignancies were hormone receptor positive; 25.7% were luminal A like, 19.3% luminal B and HER2 negative like, 7.9% luminal B and HER2-positive like, 15.7% HER2 positive, and 31.4% were triple negative.

**Conclusion:**

Women’s BC in Luanda-Angola is diagnosed at a young age and at an advanced stage. The two predominant molecular subtypes are HR positive and triple negative. The percentage of HER2-positive BC cases was high. Determining the molecular subtype using surrogate IHC markers has important treatment and prognostic implications for Angolan women with BC. There is an urgent need to study a prospective BC series in order to confirm the present results.

## Background

The cancer burden in Africa, including Angola, is likely to increase in the forthcoming decades, mostly due to the increasing life expectancy of the population, changes in lifestyles associated with economic development, and longer survival of patients with HIV-receiving antiretroviral therapy, as HIV/AIDS patients have a substantially higher risk of developing cancer than the general population [1].

Countries in sub-Saharan Africa (SSA) are currently undergoing an epidemiological transition and facing the so called ‘double-burden’ of being affected at the same time by significant infectious and chronic diseases, including malignant tumours [2].

In addition, these countries are confronted with difficulties in cancer diagnosis, stage assessment and access to cancer drugs at affordable prices and of good quality [3]. Therefore, it is crucial to carefully evaluate the profile of oncological diseases in order to avoid treating insufficiently or overtreating patients, bad practices and inadequate allocation of financial resources, while increasing survival.

Breast cancer (BC) is the second most common cancer among women in SSA; however, the incidence rate is clearly increasing [2, 4]. In SSA, BC clinical presentation differs from that in high-income countries mostly due to late-stage disease at presentation, high proportion of young patients (as the African population is predominantly young) as well as very high mortality rates [5]. In Angola, a country with 3600 doctors, 33,000 nurses, 510 pharmacists and close to 3000 health facilities serving a total population of 25.7 million (65% less than 24-year old and a life expectancy at birth for women of 63 years according to the National Statistics Institute), a similar trend is observed [6]. In Angola, most BC cases are also diagnosed at advanced stages, at a young age and present low survival rates [7, 8]. In these stages, management of BC requires an extensive and urgent approach combining actions such as surgery, timely access to cost-effective chemotherapy (cytotoxic, hormone, and target therapies) and/or radiation therapy [9]. The knowledge on BC molecular subtypes may enable more accurate diagnoses and support therapeutic decisions [10]. It is well established that depending on the molecular profile of BC, translated by IHC characterisation including advanced BC, a set of therapeutic actions should be implemented to minimise the impact of the disease [11, 12]. Molecular subtypes according to its surrogate IHC markers, classify BC into four distinct molecular subtypes: luminal A-like, luminal B-like, HER2 positive and triple negative (TN). Each subtype is associated with different targeted therapies and prognosis [13]. Hormonal treatment, for instance, is effective only in patients whose tumours express oestrogen and/or progesterone receptors (ER, PR). However, the distribution of BC hormonal phenotypes is an area of controversy in Africa. Previous studies suggest that African BC is predominantly of poor hormone receptor (HR) expression [14]. Conversely, a recent systematic review and meta-analysis reported most BC cases in Africa (including SSA) as being HR positive [15]. This discrepancy may reflect real differences, selection bias of cases and/or problems in laboratory procedures.

The dominant BC molecular profile in Angola is still unknown. To help close this knowledge gap, we conducted a preliminary study to characterise the clinical pathological profiles and molecular-like subtypes of BC in Luanda, Angola.

## Methods

### Study population

Consecutive records of the Angolan Institute of Cancer Control and *Clínica Sagrada Esperança*, Luanda – Angola, from January 2011 to 30 December 2014, were reviewed by trained doctors and information of BC cases was abstracted using a standard form. Breast tumours were staged according to the 6th edition of TNM classification [16].

### Pathology and immunohistochemistry

Histology was reviewed by expert pathologists according to the WHO 2012 classification (EF, TA, CL) [17].

A total of 179 consecutive cases of microscopically confirmed breast carcinoma were classified using IHC procedures, namely ER, PR, Ki-67 and human epidermal growth factor receptor 2 (HER2). Pre-BC systemic treatment biopsy material or surgical specimens were used for IHC.

To reduce possible laboratory constraints, duplicate IHC tests were performed in an expert laboratory in Portugal. All cases were reassessed. Of these, 39 cases (21.8%) were poorly preserved due to the low quality of the fixation and, therefore, excluded from this analysis. In total, 140 cases were processed.

Standard immunohistochemistry protocols (IHC) were performed using the following: monoclonal ER (clone 6F11, 1:150, Novocastra Laboratories, Leica Microsystems), PR (clone 16, 1:200 Novocastra Laboratories, Leica Biosystems), Ki-67/MIB-1, 1:200, DAKO (Glostrup, Denmark) and HER-2 (1:100, HercepTest DAKO) [18]. The Refine Polymer Detection kit (Leica Microsystems) was used to detect bound antibody, with 3,3-diaminobenzidine as the chromogen (Leica Microsystems). Slides were counterstained with Harris’s haematoxylin and results evaluated with positive and negative tissue controls. For ER, PR, and KI-67 antibodies only immunoreactive tumour cell nuclei were counted. Hormonal receptor evaluation was conducted according to the ASCO/CAP guidelines for immunohistochemistry [19]. Ki-67 cut-off point of 15% was defined according to the experience of different pathologists as well as national and international recommendations [20]. ER- or PR-positive nuclei greater than 1% were considered hormone receptor positive. High and very high expression of Ki-67 proliferative index was defined as nuclear expression ≥ 15% and ≥ 30% of the tumour cells, respectively.

HER2 – IHC interpretation slides were independently interpreted in a blinded fashion by two pathologists according 2013 ASCO–CAP HER2 Test Guideline Recommendations [21]. Thus, cases interpreted as 0 or 1+ were considered negative, equivocal cases (2+) were confirmed by SISH according standard procedure and cases 3+ were considered positive [22]. Cases without IHC consensus were reviewed by all pathologists at a multiheaded microscope.

Based on the immunohistochemical results, all cases were classified according to the four BC intrinsic molecular subtypes and their so-called surrogate definition (ESMO recommendations) as: *Luminal A-like* (ER+ and/or PR+, HER2−, K-67 < 15%), *Luminal B-like* (further classified according to HER2 negative: ER+ and/or PR+, HER2−, K-67 ≥ 15%; or HER2 positive: ER+ and/or PR+ HER2+), *HER2 positive* (RE− and/or RP− and HER2+) and *Triple-negative* (RE, RP and HER2 negatives) [23].

### Statistical analysis

A statistical analysis was performed using PASW Statistics for Windows, Version 18.0, 2009. Chicago: SPSS Inc. ®. Descriptive statistics are given as frequencies, median, minimum, and maximum, as interquartile range for continuous variables and as percentages for categorical variables.

### Ethics

Permission to perform this study was given by the Angolan Ministry of Health and corresponding ethics committee.

## Results

As previously mentioned, in 21.8% of the 179 studied cases, it was not possible to perform IHC studies due to poor preservation of the tissues. The distribution and clinical and pathological characteristics of the excluded cases were similar to the tested sample and did not aggregate a specific group of tumours. Consequently, a total of 140 cases were evaluated. Among these, the median age was 47 years (min. 24; max. 84 years. Percentiles 25, 50 and 75 were 39, 47 and 57 years, respectively). Invasive carcinoma of no special-type NST, also known as invasive ductal carcinoma, was the most common type (91.4%; *n* = 128) and grade-2 BC (moderately differentiated) was the most prevalent (70.7%; *n* = 99). Locally advanced carcinomas were found in 68.6% of patients (91 cases were stage III and 5 cases stage IV). In 74 malignancies (52.8%), HR were positive. Of these, 42 cases (30%) were ER and PR positive, 19 cases (13.5%) were only ER positive, and 13 cases (9.2%) were only PR positive representing 7.2% of all BC cases studied. We found HER2 immunoreactivity 3+ in 31 cases (22.1%) and 16 cases were HER2 2+. Therefore, the confirmation of amplification by SISH was performed. In only two of these 16 cases, SISH was positive. Of the 33 HER2 positive, 11 cases (7.9%) were HR positive. A total of 44 cases (31.4%) of BC were triple negative. Overall, regarding BC phenotypes, we found 63 cases (45%) HR+HER2−, 11 cases (7.9%) HR+HER2+, 22 cases (15.7%) HER2+ and 44 cases (31.4%) TN.

According to ESMO recommendations for BC molecular subtypes and their surrogate definitions, 36 cases (25.7%) were luminal A like, 27 (19.3%) were luminal B - *HER2 negative like*, 11 (7.9%) were luminal B - *HER2-positive like*, 22 (15,7%) were HER2 positive, and 44 (31,4%) were triple negative. The detailed description of the collected data is provided in Table 1.

## Discussion

Late motherhood, fewer offspring, late menopause, hormone replacement therapy, obesity and weight gain in adulthood are risk factors usually associated with an increase in HR-positive tumours in post-menopausal women [24]. However, in SSA, these risk factors are not frequent. On the other hand, low socio-economic status, young age at diagnosis, and BRCA1 mutation are more strongly associated with HR-negative tumours, and somewhat closer to SSA reality [25]. Nevertheless, BC molecular subtype’s dominant distribution in African populations is still unclear.

To the best of our knowledge, this is the first report on receptor-defined subtypes of BC in indigenous populations of Angola and it is a contribution to clarify the SSA profile of BC receptor status.

Angola is characterised by a high prevalence of late stage BC at diagnosis, which occurs at young age. As observed in most SSA countries invasive carcinoma (NST) and high histologic grade are predominant [5, 26].

In our study, relatively high rates of TN and HER2-positive cases were identified; however, HR+/HER2− phenotype was predominant, similarly to what happens in the African–American population (Figure 2) [27]. According to the 2015 St Gallen Consensus Conference and ESMO surrogate definitions of BC molecular subtypes, in our series, 25.7% were luminal A like, 19.3% were luminal B - HER2-negative like, 7.9% were luminal B - HER2-positive like, 15,7% were HER2 positive and 31,4% were triple negative. The rate of oestrogen receptor negative/progesterone receptor- ositive was 7.2% of all BC cases studied, somewhat higher than is usually described. Several previous studies have shown ER negative/PR positive class constitutes 2–7% of breast cancer [28]. Shen T *et al*. found that the ER/PR+ rate was higher in African Americans than in the white population, these patients were young and had high-grade and aggressive tumours [29]. Our cases were predominantly high grade and occurred in young pre-menopausal women similar to that recorded by others [29, 30]. Immunohistochemical studies were repeated and we had consistent results. However, De Maeyer *et al*. indicates that the use of different thresholds for positivity and technical sensitivity in the different studies may obscure the results and their comparison [28, 31]. Consequently, further confirmatory studies are recommended. To evaluate our results, we have thoroughly reviewed the studies carried out in SSA.

Thereby, McCormack V *et al*. studied 1200 South African patients and found that the majority of tumours were ER positive in black patients, triple-negative cases constituted one fifth of tumours, black women were more likely than non-black women to have ER negative or triple negative, and the HER2+ proportions were relatively high [32]. Dickens C *et al*. studied ER, PR and HER2 receptor status in two multiracial Southern African countries (South Africa and Namibia) with routine diagnostic IHC and concluded that positive ER breast cancer dominates in all Southern African races [33]. However, different results were observed in other regions of Africa. For instance, Titloye *et al*. studied a total of 835 tumours from Nigerian patients and the predominant molecular phenotype found was triple-negative type (47.6%) followed by the HER2-positive group (19.6%) [34]. Rambau P *et al*. found, in North-Western Tanzanian BC patients, a low level of expression of HR and a significant proportion of triple negative [35]. Luyeye Mvila G *et al*. compared a BC series diagnosed in Kinshasa (Africans) with a European series of BC diagnosed in Leuven and found that the first group presented BC at younger ages and mainly ER-negative and HER2-positive tumours, when compared to the Caucasian group. However, no difference in the rate of triple-negative BC was observed [36]. Ly M *et al*. found a high incidence of aggressive triple-negative tumours in Mali [37]. A study comprising 507 patients diagnosed with breast cancer between 1996 and 2007 at six geographic regions in Nigeria and Senegal showed that 76% of cases were ER negative [38]. In Lusaka, Anastase Nkuliyingoma (2015) studied 46 patients with BC and found ER negative in 54.3%, PR negative in 47.8% and 4.3% HER2 positive [39]. In Ghana, divergent results were observed. Adjei EK *et al*. studied 51 BC cases, of which 76% were ER+ ( ≥ 1% ER+ tumour cells) and 22% were triple negative [40]. However, in Kumasi, BC affects mostly young pre-menopausal women who presented advanced disease. Of the 54 BC cases studied, 47%, 13.2%, and 20.2% were positive for ER, PR, and HER2 (3+), respectively, and 42.7% were triple negative [41].

**Figure 1.**
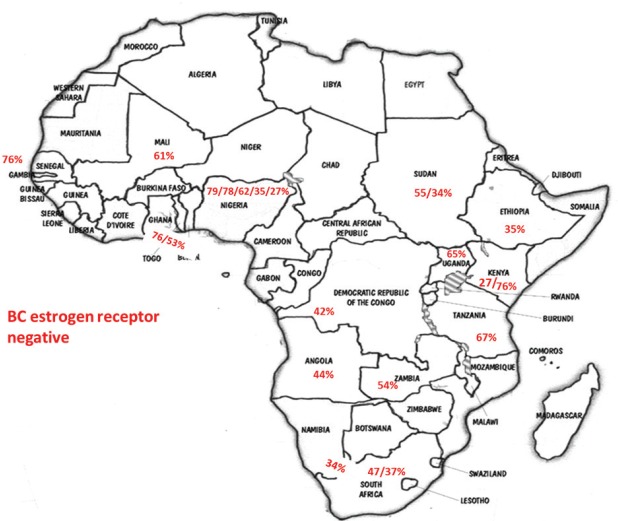
Percentage of negative oestrogen receptor in different SSA countries according to the data of studies referenced in this paper as well as our results (BC – breast cancer).

**Figure 2.**
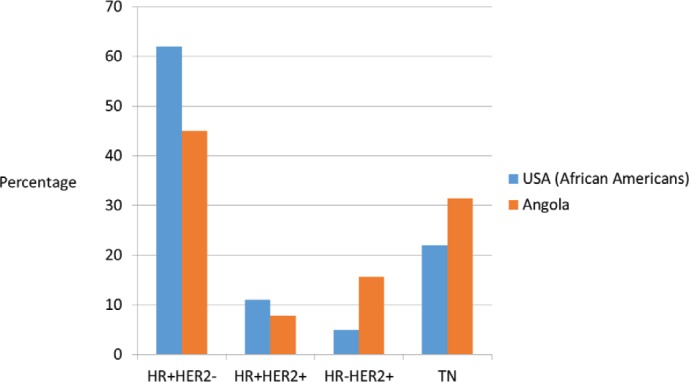
Distribution of breast cancer phenotype in United States African–American population [27] and Angola population (our study).

With the aim of clarifying these differences, Jamal and Fedewa reported that the prevalence of BC-negative HR in US-born black women was similar to BC in African patients from Western Africa living in the USA and in Caribbean black women. The prevalence of BC-negative HR was substantially lower in BC of black women born in East Africa and living in the USA [42]. Curiously, Kantelhardt EJ *et al*. verified that only 35% of the cases were ER negative in Ethiopia (an East African country) and the proportion of ER-negative tumours decreased with advancing age at diagnosis and was not affected by histology or stage [43]. Corroborating this data, a study conducted in Kenya (an East African country) by Syed S *et al*. presented a definitive prospective analysis of ER/PR/HER2 from a single centre and demonstrated that the prevalence of receptor status was comparable with that in the West [44]. Our results and abovementioned studies regarding ER negative were aggregated in Figure 1. In Table 2, we compare our results with the available SSA breast cancer phenotype data [32–34, 36, 38, 44–46]. We found that results from Luanda, Angola are quite similar to those described in neighbouring countries.

Given the above, could the diversity found between West and East Africa regarding HR and HER2 expression rate in BC be also explained by the African population migrations? [47, 48]. Do these migration aspects have any influence in our results? Is it reasonable to hypothesise that poor survival in SSA countries may also be related to the presence of indigenous poor prognostic factors? There is no doubt that Ethno-oncology is a challenging field to be developed in the future. Molecular markers of breast cancer should be investigated in multiethnic populations to determine their role as therapy targets, predictors or prognostic indicators of disease [49]. As pointed out by Eng A *et al*. no single-molecular BC subtype dominates in Africa [15]. Consequently, receptor testing availability should be a priority, in order to offer the best BC treatment modalities.

Finally, some methodological limitations of the present study should be mentioned. Due to sample size constraints and the absence of data provided by other municipalities in Angola, the presented results may not be representative of the Angolan female population with BC and results should only be generalised to women treated in Luanda. Another aspect to be noted refers to the fact that 39 cases (21.8%) were poorly preserved. Nonetheless, the distribution of these cases was random and did not aggregate a specific group of tumours, and therefore did not compromise the obtained results.

## Conclusion

Women with BC in Luanda-Angola are diagnosed at a young age and at an advanced stage. The two predominant molecular subtypes are HR positive and triple negative. The percentage of HER2-positive BC cases was high. Determining the molecular subtype using surrogate IHC markers has important treatment and prognostic implications for Angolan women with BC. There is an urgent need to study a prospective BC series in order to confirm the present results in a more representative sample of the Angolan population.

## Conflicts of interest

No conflicts of interest to disclose. No financial or commercial relationships.

## Authors’ contributions

This study was conceptualised, designed, and written by Lúcio Lara Santos. Acquisition of data was carried out by Fernando Miguel, Lygia Vieira Lopes, Emilia Ribas, Alexis Fuentes Pelaez, Paula Lopes and Lúcio Lara Santos. Analysis, and interpretation of data were done by Eduardo Ferreira, Teresina Amaro, Carlos Lopes, Conceição Leal, Cristina Santos and Lúcio Lara Santos. Fernando Miguel, Lygia Vieira Lopes, Eduardo Ferreira, Emília Ribas, Alexis Fuentes Pelaez, Conceição Leal, Teresina Amaro, Paula Lopes, Cristina Santos, Carlos Lopes, and Lúcio Lara Santos drafted or revised the article for important intellectual content. All authors read and agreed to the final version of this manuscript. Fernando Miguel, Eduardo Ferreira, Lygia Vieira Lopes, and Lúcio Lara Santos equally contributed to this study.

## Figures and Tables

**Table 1.** Association of BC molecular subtypes according to its surrogate IHC markers and clinicopathologic features.

Variable	Luminal-like A*	Luminal-like B*		HER2 positive*	Triple-negative*
		HER2 negative	HER2 positive		
**Number of cases (%)**	36 (25.7)	27 (19.3)	11 (7.9)	22 (15.7)	44 (31.4)
**Age (years)** Median Minimum Maximum	48.5 29 78	48.0 24 84	41.0 30 68	41.0 28 71	47.5 30 83
**Histologic type** Infiltrating ductal carcinoma Infiltrating lobular carcinoma Metaplastic carcinoma Papillary carcinoma Intracystic papillary carcinoma	32 1 1 1 1	26 1 0 0 0	11 0 0 0 0	22 0 0 0 0	40 3 1 0 0
**Grade** 1 2 3	5 27 4	3 22 2	0 6 5	0 13 9	0 26 18
**Stage** I IIa IIb IIIa IIIb IIIc IV NA	0 3 9 11 5 5 2 1	0 3 2 12 6 1 1 2	0 1 1 3 4 1 1 0	1 2 3 6 5 1 1 3	0 3 8 17 13 1 0 2
**Hormone receptors** Positive Negative	36 0	27 0	11 0	0 22	0 44
**Ki-67** < 15% 15–30% > 30%	36 0 0	0 24 3	0 8 3	8 11 3	27 13 4


* According ESMO recommendations [22].

NA – Not announced.

**Table 2.** Results of studies with data on surrogate molecular breast cancer subtypes in indigenous populations in sub-Saharan Africa.

Study	Country	Luminal A-like number (%)	Luminal B-like number (%)	HER2+ number (%)	Triple–negative number (%)
Huo D *et al* [38]	Nigeria and Senegal	102 (37.7)	9(3.32)	57 (21.03)	103 (38.0)
Agboola AJ *et al* [45]	Nigeria	48 (26.1)	10 (5.4)	34 (18.5)	69 (37.5)
Titloye NA *et al* [34]	Nigeria	70 (15.0)	26 (5.0)	92 (20.0)	279 (48.0)
Adebamowo CA *et al* [46]	Nigeria	118 (77.6)	4(2.6)	6 (4.0)	24 (15.8)
Luyeye MG *et al* [36]	Democratic Republic of Congo	54 (62.07)	13 (14.94)	7 (8.05)	13 (14.94)
**Our study**	**Angola**	**63 (45.0)**	**11 (7.9)**	**22 (15.7)**	**44 (31.4)**
Dickens C *et al* [33]	South Africa and Namibia	2416 (54.6)	610(13.8)	474 (10.7)	925 (20.9)
McCormack VA *et al* [32]	South Africa	551 (53.7)	150 (14.6)	117 (11.04)	209 (20.4)
Sayed S *et al* [44]	Kenya	175 (61.2)	31 (10.8)	22 (7.7)	58 (20.2)

**Luminal A-like** (HR+HER2−); **Luminal B-like** (HR+HER2+); **HER2+** (HR−HER2+) and **TN** (HR−HER2−). **HR** (hormone receptors), **HER2** (HER 2 receptors) and **TN** (triple negative).
